# Cardiac Magnetic Resonance Features of Fabry Disease: From Early Diagnosis to Prognostic Stratification

**DOI:** 10.31083/j.rcm2305177

**Published:** 2022-05-16

**Authors:** Antonia Camporeale, Alberto Diano, Lara Tondi, Silvia Pica, Giulia Pasqualin, Michele Ciabatti, Francesca Graziani, Maurizio Pieroni, Massimo Lombardi

**Affiliations:** ^1^Multimodality Cardiac Imaging Section, IRCCS Policlinico San Donato, San Donato Milanese, 20097 Milan, Italy; ^2^Department of Clinical Science and Community Health, University of Milan, 20122 Milan, Italy; ^3^Department of Cardiology, San Donato Hospital, 52100 Arezzo, Italy; ^4^Department of Cardiovascular and Thoracic Sciences, Fondazione Policlinico Universitario A. Gemelli IRCCS, 00168 Rome, Italy

**Keywords:** fabry disease, cardiac magnetic resonance, late gadolinium enhancement, T1 mapping

## Abstract

In the past few years, the wide application of cardiac magnetic resonance (CMR) significantly changed the approach to the study of cardiac involvement in Fabry Disease (FD). The possibility to perform non-invasive tissue characterization, including new sequences such as T1/T2 mapping, offered a powerful tool for differential diagnosis with other forms of left ventricular hypertrophy. In patients with confirmed diagnosis of FD, CMR is the most sensitive non-invasive technique for early detection of cardiac involvement and it provides new insight into the evolution of cardiac damage, including gender-specific features. Finally, CMR multiparametric detection of subtle changes in cardiac morphology, function and tissue composition is potentially useful for monitoring the efficacy of specific treatment over time. This paper aims to provide a comprehensive review of current knowledge regarding the application of CMR in FD cardiac involvement and its clinical implication.

## 1. Introduction

Fabry Disease (FD) is a rare X-linked lysosomal storage disorder, characterized by abnormally low or absent alpha galactosidase A activity, leading to intracellular glycosphingolipid accumulation in many organs and tissues [1]. Due to FD X-linked transmission, men are generally more affected than women, who may present with variable clinical pictures [2]. Heart involvement represents an important cause of morbidity and mortality, mainly due to malignant ventricular arrhythmias and heart failure [3]. Fabry cardiomyopathy results from progressive glycosphingolipid storage in all cardiac cell types, leading to left ventricular hypertrophy (LVH), myocardial fibrosis and/or inflammation. Such myocardial alterations may produce progressive left ventricular (LV) diastolic and systolic dysfunction, microvascular ischemia, brady- and tachyarrhythmias [4]. Cardiac involvement in FD may mimic the morphological features of sarcomeric hypertrophic cardiomyopathy (HCM) and the differential diagnosis between these two clinical entities is challenging, especially in patients without extracardiac FD manifestations (the so-called “cardiac variant”) [5]. The importance of the differential diagnosis between FD cardiomyopathy and other forms of myocardial hypertrophy is related to the availability of specific treatment for FD, namely two different formulations of Enzyme Replacement Therapy (ERT) [6, 7] and a pharmacological chaperone [8]. To optimize the effect of these therapies, early identification of FD cardiac involvement is pivotal to preventing the occurrence of irreversible organ damage. Recently, cardiac magnetic resonance (CMR) has gained increasing importance in the evaluation of patients with known or suspected FD, thanks to its capability to provide crucial information for differential diagnosis, early detection, prognostic stratification, and follow-up of heart involvement [9]. The wide application of CMR in the study of FD patients has also provided new insight into gender differences in phenotypic expression [10]. This paper aims to offer a comprehensive review of current knowledge regarding the application of CMR for the study of FD cardiac involvement and its clinical implication.

## 2. Basic CMR Principles

Compared to echocardiography, CMR is more accurate for quantification of LV volumes, ejection fraction, and mass, both in normal and pathologic hearts [11]. Indeed, CMR directly measures these parameters by the acquisition of cine images in contiguous slices, thus limiting geometric assumptions. Different views of the organ can be obtained with high spatial resolution and sharp contrast between blood and myocardium, without limitation related to the acoustic window. Thanks to these features, CMR is the ideal tool for the identification of borderline forms of LVH, the quantification of myocardial trabeculation, the detection of segmental LVH patterns, and the monitoring of small changes in LV mass over time.

The myocardial feature tracking technique can be applied to cine images to quantify atrial and ventricular deformation, similar to speckle tracking echocardiography [12, 13].

Moreover, CMR offers the unique possibility to non-invasively describe tissue composition. Late Gadolinium enhancement (LGE) is the milestone of CMR tissue characterization, allowing the detection of an increase in extracellular space, often due to myocardial edema and/or fibrosis. The LGE pattern provides crucial information about the pathophysiology of myocardial damage, distinguishing ischemic from non-ischemic causes [14].

**Fig. 1.**
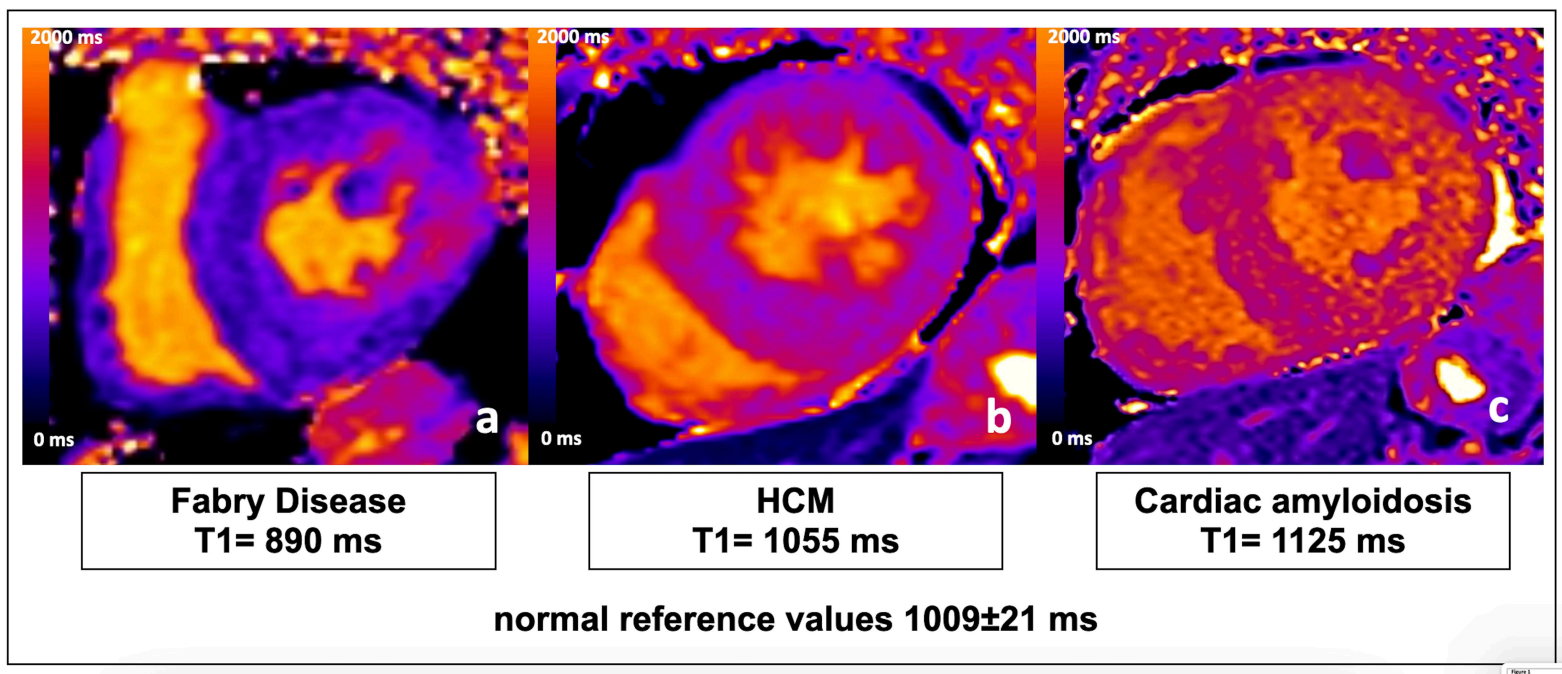
**Mid-ventricular short axis view of T1 maps (modified Look-Locker inversion recovery (MOLLI) sequence) in a patient with Fabry Disease (a), sarcomeric HCM (b), and cardiac amyloidosis (c)**. The difference in native septal T1 values (T1 reduction in Fabry disease, normal T1 in HCM, and markedly increased T1 in cardiac amyloidosis) allowed differential diagnosis.

T1 and T2 mapping represent the new frontiers of CMR tissue characterization [15]. Changes in myocardial relaxometric properties (T1-longitudinal relaxation time and T2-transverse relaxation time) reflect focal or diffuse alterations in tissue composition that can be measured and visualized in color-encoded maps, in which the pixel values represent the T1/T2 in each voxel. In particular, T1 mapping sequences allow quantification of two important parameters: native (pre-contrast) T1 and extracellular volume fraction (ECV). Native T1 increases in presence of edema and protein accumulation, while it decreases in case of lipid or iron deposition. In FD-related LVH, myocardial glycosphingolipid storage leads to a lowering of native T1 compared to normal reference values and to other forms of LVH such as HCM or cardiac amyloidosis, thus helping in the differential diagnosis (Fig. 1) [16]. ECV estimation derives from the acquisition of both pre- and post-contrast T1 mapping sequences and detects slight and diffuse increases in extracellular space, even when LGE images are negative.

T2 mapping can directly quantify local myocardial inflammation and edema and found wide application in the study of myocarditis [17]. Increased T2 in the LGE positive area has been reported in FD cardiomyopathy, with higher values compared to HCM and chronic myocardial infarction.

It must be specified that both T1 and T2 mapping are strongly influenced by several factors including scan vendor, type of sequences, and post-processing software. Thus, each center needs to refer to internal reference values. Caution is advised when comparing T1/2 values from different centers if the scanner configuration and post-processing system are not identical and proper quality controls have not been performed.

**Fig. 2.**
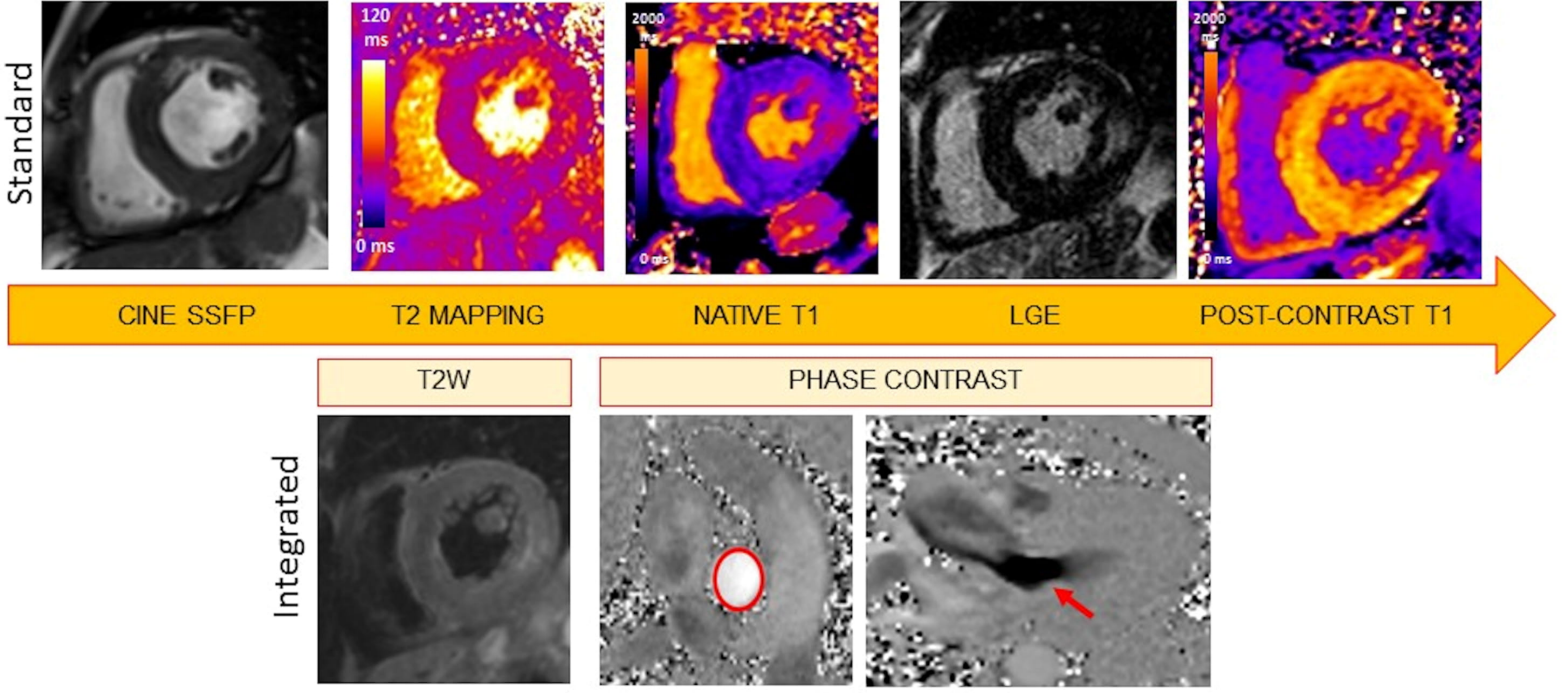
**Suggested CMR protocol for accurate morphological, functional, and tissue characterization of FD cardiomyopathy**. Cine images, T2 mapping, pre and post-contrast T1 mapping with ECV quantification, and LGE images should always be included. This standard protocol can be implemented with T2w images covering the whole heart and phase contrast images in suspicion of left ventricular outflow obstruction (red arrow). The red circle identifies the proximal ascending aorta. Measurement of the aortic flow allows the quantification of the degree of SAM-related mitral regurgitation.

A CMR study in patients with known or suspected FD should include all these sequences (cine, LGE, T1, and T2 mapping), to provide a comprehensive and detailed evaluation of cardiac morphology and function, with multiparametric tissue characterization (Fig. 2).

This standard CMR protocol can be implemented by adding T2 weighted images covering the whole heart and Phase Contrast images. The last Consensus Statement by the Society of Cardiovascular Magnetic Resonance and the European Association of Cardiovascular Imaging on clinical recommendations for CMR relaxometric mapping [15] suggested the acquisition of specific T1/2 mapping slices according to the disease. T2 weighted images covering the entire short axis stack can help identify areas of increased myocardial signal indicating edema in atypical locations that can be missed by standard cuts. Several cases of FD patients with obstructive LVH (both outflow tract obstruction and mid-ventricular obstruction) have been described in previous literature [18, 19]. The acquisition of Phase Contrast images in FD patients with suspected LV obstruction can identify the site of the obstruction (in-plane 3 chambers view) and eventually quantify flow velocity (through-plane orthogonal to the site of the obstruction). Measurement of the aortic flow allows the quantification of the degree of Systolic Anterior Motion (SAM)-related mitral regurgitation [20].

## 3. LV Morphology and Function 

### 3.1 LV Morphology

**Fig. 3.**
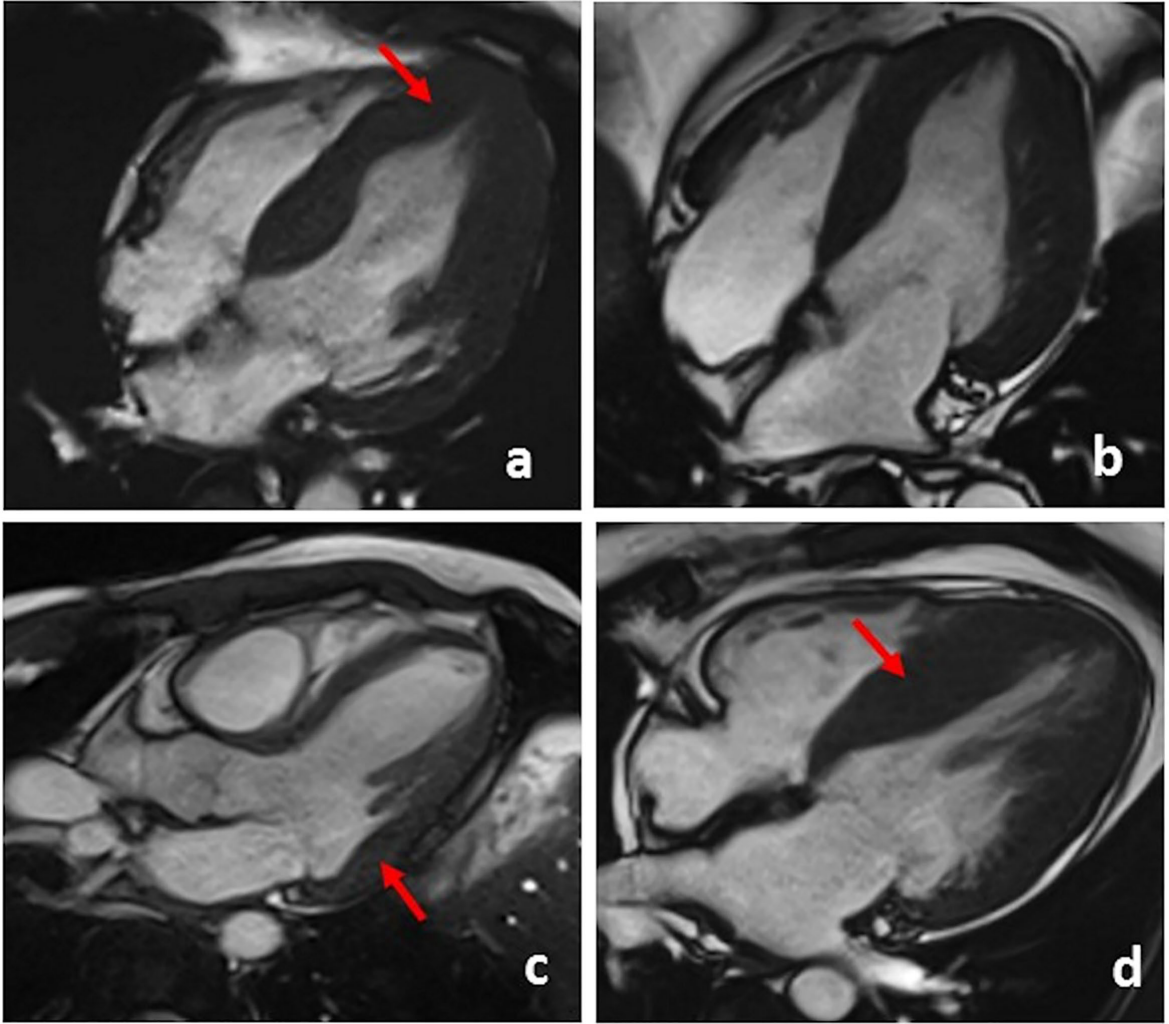
**Cine images showing different patterns of LVH in Fabry patients**. (a) LVH with apical involvement. (b) Global LVH. (c) Focal LVH of the basal infero-lateral wall. (d) Asymmetric septal LVH.

The hallmark of FD cardiomyopathy is concentric wall thickening and the severity of LVH is related to the risk of ventricular arrhythmias [4]. In particular, LV mass index assessed by CMR is a better predictor of adverse cardiovascular outcomes compared to LV mass index assessed by 2D echocardiography [21]. In females, LVH onset is delayed by 10 years compared with males and its degree is generally less severe [22]. The increasing application of CMR unraveled a wide spectrum of morphological phenotypes of FD cardiomyopathy beyond concentric LVH (Fig. 3). In a population of 39 FD patients, Deva *et al*. [22] described a subgroup of 5 patients with asymmetrical and apical hypertrophy, showing greater maximum wall thickness, total LV scar, apical and mid-ventricular scar than patients with concentric hypertrophy (n = 17). Thus, the pattern of LVH should not be considered a discriminating element in the differential diagnosis between FD and other causes of LVH.

Increased myocardial trabeculation has been reported in FD cardiomyopathy by both echocardiography and CMR. CMR allows the quantification of the degree of myocardial trabeculation as a percentage of total LV mass or by fractal analysis, describing the complexity of the endocardial border. Kozor *et al*. [23] demonstrated that papillary muscle and trabecular mass contribute to an average of 20% of the total LV mass in a group of 20 FD male patients, over one and a half times more than the control group. Failure to account for trabeculations during LV segmentation may result in significant underestimation of LV mass and lead to misclassification of a proportion of subjects. Trabecular complexity measured by fractal analysis has been shown to parallel the evolution of the cardiac phenotype of FD, with a progressive increase in more advanced stages [24].

Other morphologic abnormalities previously described in sarcomeric HCM have also been reported in FD cardiomyopathy, namely myocardial crypts and increased length of the anterior mitral valve leaflet [25, 26]. Our group showed that these anomalies progressively increase with the severity of FD cardiomyopathy, as well as myocardial trabeculation.

### 3.2 LV Function and Myocardial Deformatin 

Most studies regarding myocardial deformation in FD have been performed using speckle tracking echocardiography, while only a few recent works applied CMR feature tracking. Both imaging techniques agreed in reporting that LV ejection fraction is usually preserved until advanced disease stages, while the presence of LVH and/or LGE is associated with impairment of LV global longitudinal strain (GLS). In particular, Krämer *et al*. [27] previously demonstrated that the loss of global deformation, quantified by speckle tracking, was predominantly caused by LGE positive segments, namely the basal posterior and the lateral, and that GLS correlated with the amount of LGE. Lowering of myocardial T1 values and the presence of electrocardiogram (ECG) abnormalities have also been associated with impairment in GLS in a previous study by Vijapurapu *et al*. [28], including 221 FD patients. In a recent longitudinal CMR study by Nordin *et al*. [29], worsening of GLS at one-year follow-up paralleled the increase in T2 value measured in the LGE area. While the impairment of GLS tracks the advanced stages of the disease, the loss of base-to-apex circumferential strain (CS) gradient has also been reported in the absence of hypertrophy or LGE, allowing discrimination from healthy controls independently of native T1 [30].

### 3.3 Morphological LV Changes in Early Disease Stage

**Fig. 4.**
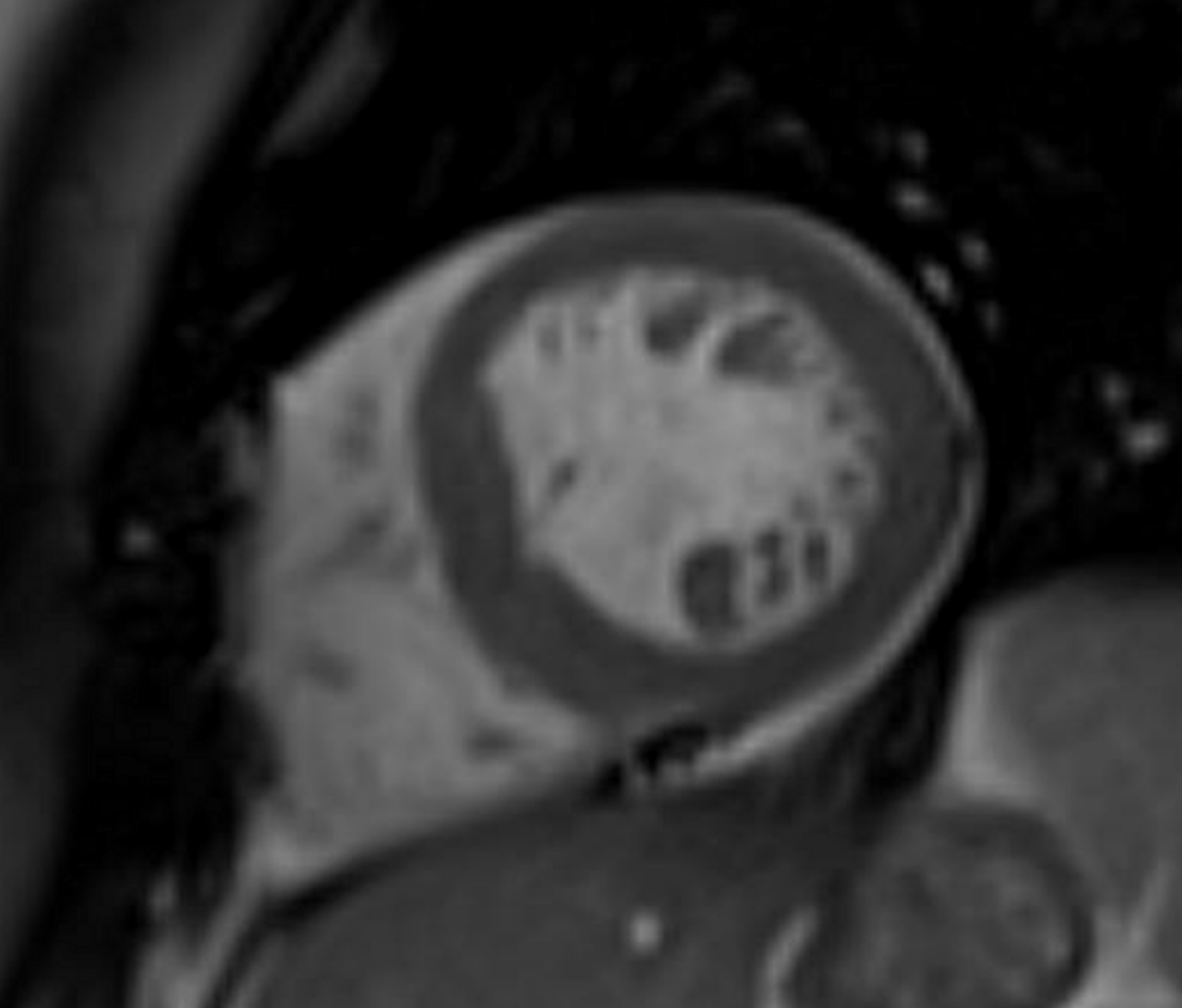
**Example of increased LV trabeculation in a 44 year-old female FD patient, in the absence of overt LVH and LGE**.

According to a recent model of FD cardiomyopathy evolution, a pre-hypertrophic phenotype can be recognized before the occurrence of LVH, myocardial inflammation and fibrosis [30]. This early phase, defined as the ‘accumulation phase’, is characterized by a progressive increase in myocardial glycosphingolipid storage and lowering of native T1 value, paralleled by a growing increase in LV mass and LV wall thickness. Among pre-hypertrophic FD patients, the presence of low myocardial native T1, indicating myocardial glycosphingolipid storage, has been reported in 59% of patients and represents a risk factor for disease worsening at one-year follow [31]. Moreover, low myocardial T1 values in LVH negative FD patients are associated with early morphological alterations, some of which were previously described in genotype positive/phenotype negative HCM patients [25]. Increased myocardial trabeculation, increased length of the anterior mitral valve leaflet, increased number of myocardial crypts [24], higher frequency of ECG abnormalities [32] and lower stress myocardial blood [33] flow have been reported in LVH negative/low T1 patients, as compared to LVH negative/normal T1 FD patients (Fig. 4). Recently, some very early alterations, such as greater myocardial trabeculation, early impairment in stress myocardial blood flow and subtle ECG abnormalities, have also been reported in LVH negative/normal T1 patients compared to healthy controls, thus defining a “pre-storage” phenotype [34].

## 4. LV Tissue Characterization 

### 4.1 Late Gadolinium Enhancement

LGE was first described in both male and female patients with FD by Moon *et al*. in 2003 [35]. Three years later, the same Authors published a histological study demonstrating that LGE is caused by focal myocardial collagen scarring [36]. According to this finding, LGE in FD cardiomyopathy is considered a sign of irreversible organ damage.

**Fig. 5.**
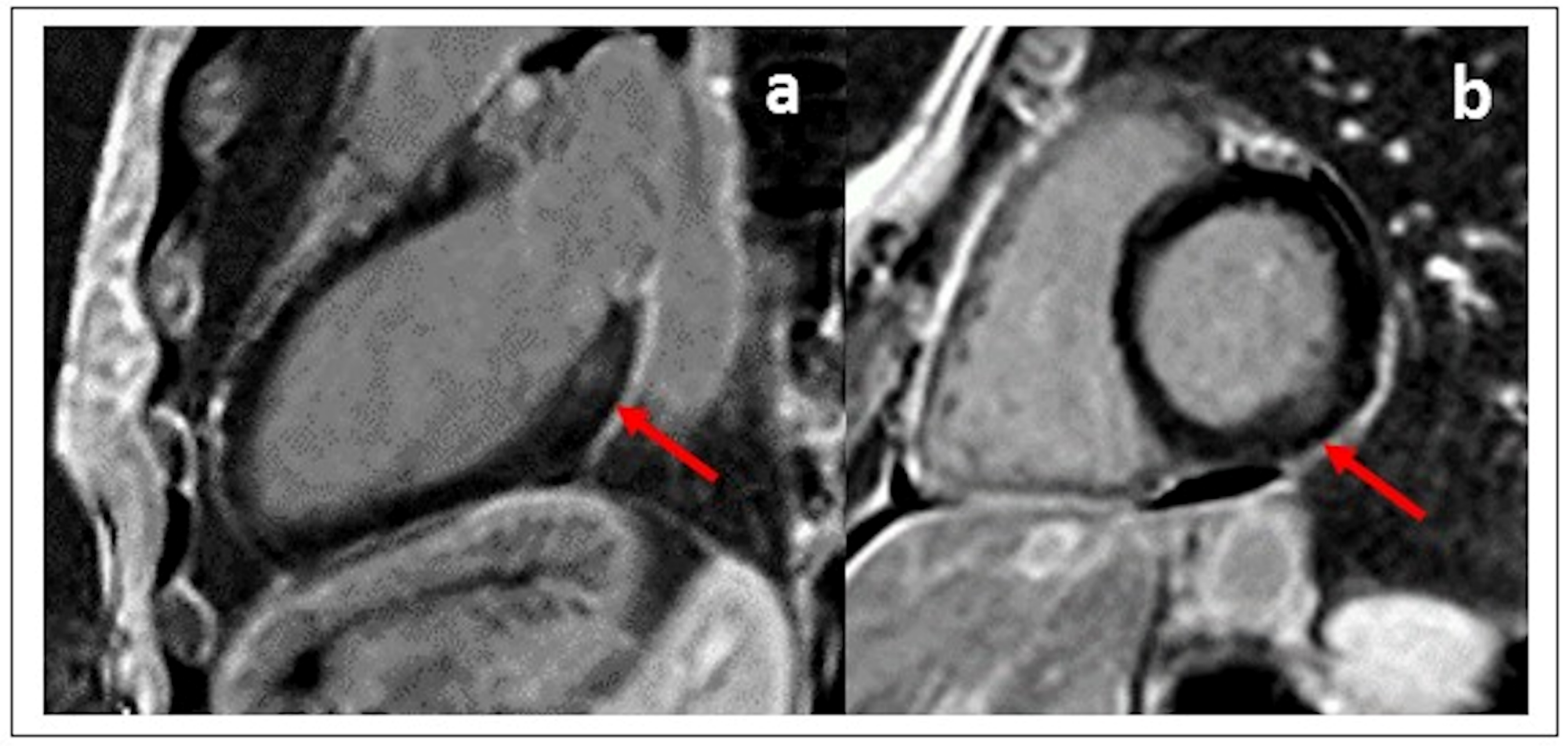
**LGE images ((a) vertical long axis, and (b) basal short axis) in a female FD patient without left ventricular hypertrophy showing intramyocardial accumulation of contrast medium (non-ischemic pattern) in the basal infero-lateral wall**.

In the majority of patients (about 75%), LGE is located in the basal infero-lateral wall with mid wall pattern [37], but other atypical locations have also been described [22]. The reasons for such location and distribution are still unclear and it has been suggested that they may reflect inhomogeneous LV wall stress. LGE prevalence is higher in men than women (59% vs. 37%) and increases with age in both sexes. In FD male patients there is a positive association between LV mass and LGE, while in females it is common to find LGE even without LVH (Fig. 5) [10]. Thus, CMR evaluation in FD female patients is pivotal to detect advanced cardiac damage missed by echocardiographic assessment.

In FD LGE represents a risk factor for sudden cardiac death together with LVH, age, male gender, and non-sustained ventricular tachycardia [38, 39]. Moreover, the presence of LGE has been associated with poor response to ERT [40, 41].

### 4.2 T1 Mapping

More than 90% of LVH-positive FD patients showed reduced myocardial native T1, allowing to distinguish FD from all other common causes of LVH [15]. In LVH negative FD patients T1 inversely correlates with LV mass in both genders (the lower the T1, the greater the LV mass). Interestingly the relationship between T1 and LV mass develops gender-related differences at the occurrence of LVH [29]. In male patients with overt LVH, there is a positive correlation between T1 and LV mass likely due to cardiomyocytes hypertrophy triggered by storage. Conversely, among LVH-positive female patients, there is no significant correlation between LV mass and T1. A recent histological study by Chimenti *et al*. [42] showed a mosaic of affected and unaffected cardiomyocytes in myocardial specimens from 24 FD female patients. Unaffected myocytes’ size correlated with maximum LV wall thickness and their contribution to determining LVH could explain the sex dimorphism in the relationship between T1 value and the degree of LVH.

**Fig. 6.**
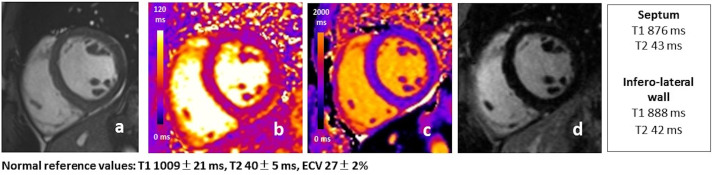
**Example of accumulation phase: 27 year-old male FD patient with classic mutation showing reduced native T1 (c) in the absence of LV hypertrophy (a), myocardial inflammation (b), and LGE (d)**. T1 and T2 values measured in the interventricular septum and the infero-lateral wall are reported on the side.

The only CMR study including evaluation of children with FD (n = 15) showed that all patients were LVH-negative with normal LV function and native T1, and that T1 fell linearly with increasing age [29]. As concerns clinical implications of T1, Réant *et al*. [43] demonstrated that low myocardial T1 predicts de novo atrial fibrillation or TIA/stroke in a population of 35 FD patients. However, the strongest clinical impact of native T1 in FD relays on the early detection of cardiac involvement (Fig. 6) [44, 45]. Our group and other Authors described several initial morpho-functional alterations associated with T1 reduction in the pre-hypertrophic phase (see previous paragraph ‘*Morphological LV changes in early disease stage*’). In particular, ECG tracks low T1 value better than other imaging techniques since shorter PR interval corrected for heart rate, longer P wave duration, shorter PR segment, lower P wave/PR segment ratio, greater Sokolow Lyon Index and T wave amplitude have been reported in LVH negative-low T1 patients compared to LVH negative-normal T1 patients [46]. Reduced T1 values have also been associated with systemic disease worsening at one-year follow-up [30]. Taken together, all these data led the experts to indicate low myocardial T1 as an early cardiac disease marker to be considered to target therapeutic strategies [47]. Native T1 currently represents the only non-invasive tool to detect myocardial storage, however, the sensitivity and specificity of T1 mapping in FD have never been evaluated in comparison with histological findings.

**Fig. 7.**
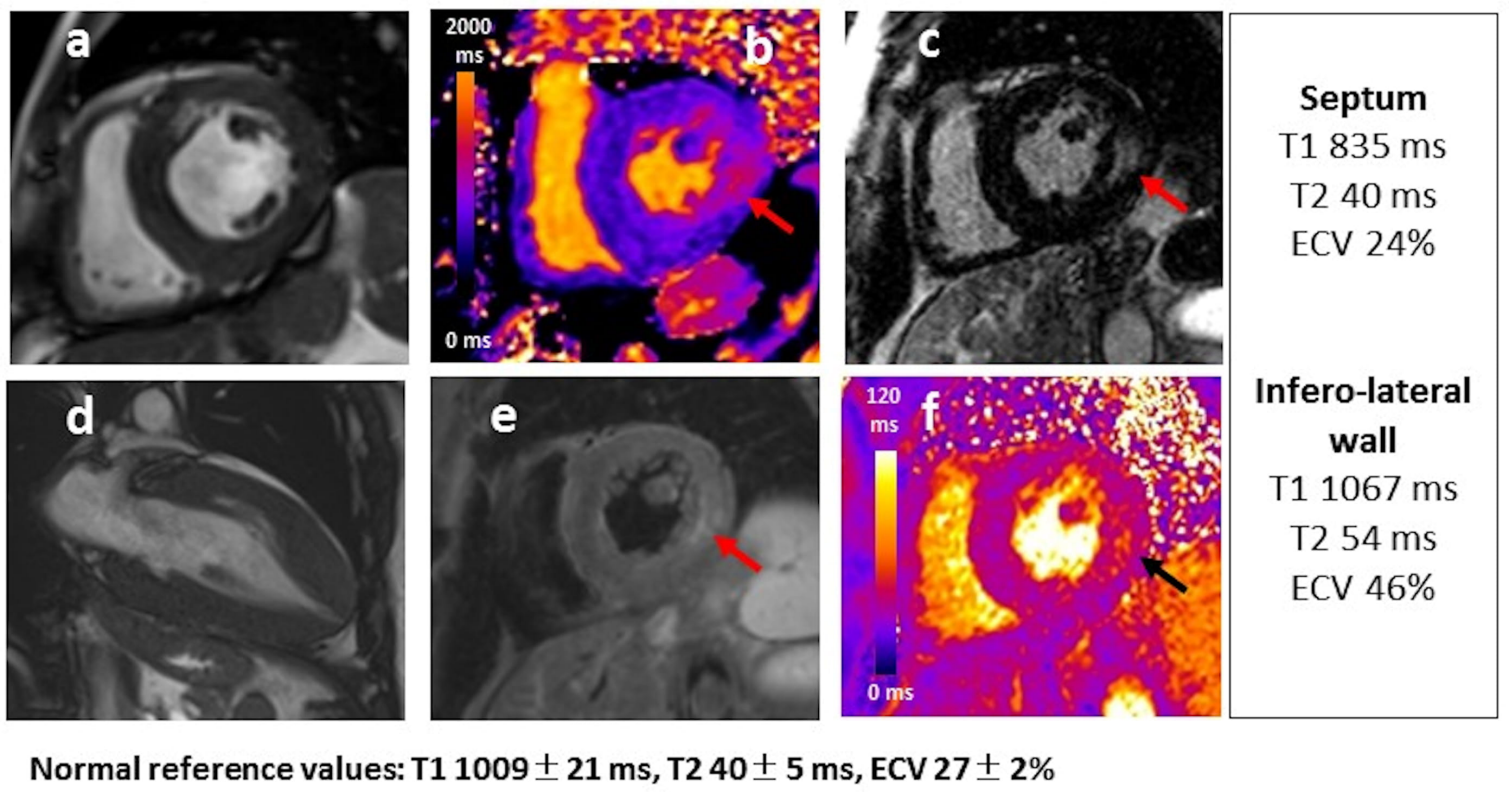
**Advanced FD cardiomyopathy: 52 year-old male FD patient with classic mutation showing LVH with apical involvement in cine images (a,d), myocardial inflammation of the infero-lateral wall with hyperintense signal in T2w sequences (e) and increased T2 values (f), intramyocardial fibrosis of the infero-lateral wall with increased native T1 (b) and LGE (c)**. T1, T2 and ECV values measured in the interventricular septum and the infero-lateral wall are reported on the side.

ECV is usually within the normal range in FD apart from the LGE positive areas. The presence of LGE is associated with pseudonormalization or elevation of T1 values paralleled by an increase in ECV (Fig. 7). Indeed, increased spread of segmental myocardial T1 values between myocardial segments with glycosphingolipid accumulation (low native T1) and segments with fibrosis, or inflammation (high native T1) has been proposed as a biomarker of cardiac involvement in FD across the disease severity spectrum [48]. According to the current knowledge, ECV does not play any specific diagnostic or prognostic role in FD.

### 4.3 T2 Mapping

Increasing evidence suggests that glycosphingolipid accumulation in FD cardiac cells can generate a pro-inflammatory response. Nordin *et al*. [49] previously reported a marked increase in T2 values in LGE positive areas in FD patients. In multivariate analysis, T2 in the basal infero-lateral wall was the strongest predictor of an increase in troponin level. According to these findings, the Authors suggested that FD with LGE is not only a storage disease but also a chronic inflammatory cardiomyopathy. Moreover, Augusto *et al*. [50] recently demonstrated that high basal infero-lateral wall T2 predicted clinical worsening at 1-year follow-up. The presence of myocardial inflammation in FD cardiomyopathy has been confirmed by hybrid Positron Emission Tomography (PET)/Magnetic Resonance (MR) imaging, showing focal Fluorodeoxyglocose (FDG) uptake in LGE/T2 positive myocardial segments in a population of 13 patients [51]. Finally, Frustaci *et al*. [52] recently provided histological evidence of immune-mediated myocarditis correlated with disease severity in endomyocardial biopsies from 78 patients with FD cardiomyopathy. The role of myocardial inflammation in the evolution of cardiac damage and its interaction with specific disease treatment remains to be elucidated.

## 5. RV and LA Involvement

Besides the description of the well-known LV involvement in FD, autopsy studies showed the deposition of globotriaosylceramides also in the right ventricle (RV) and the left atrium (LA), confirming that FD cardiomyopathy is a pan cardiac disease [53].

In previous echocardiographic studies, RV hypertrophy emerged as a common finding in patients with FD and correlated with disease severity and LVH [54, 55]. However, unlike cardiac amyloidosis, RV hypertrophy does not significantly affect RV systolic function and does not influence prognosis [56]. So far, no CMR studies have systematically studied RV morphology and function in FD. Regarding RV tissue characterization, RV LGE has never been reported in FD, while reduced native T1 values have been measured in a small population of FD patients with RV wall thickness >4 mm [57].

Regarding LA involvement, our group performed a systematic evaluation of LA volumes and function by CMR feature tracking in a population of 45 FD patients, stratified according to the degree of LV involvement [58]. Atrial deformation was already impaired in LVH negative patients with low T1 and normal diastolic function, and a good correlation was found between LA total strain and native T1 values. This finding supported the concept of atrial myopathy directly caused by glycosphingolipid deposition and introduced LA total strain as a potential novel indicator of early cardiac involvement. The atrial myopathy appeared to progress in parallel with ventricular features of FD cardiomyopathy as well as with extracardiac manifestations.

## 6. Differential Diagnosis with Other Forms of LVH

Since FD may potentially benefit from specific therapies, the differential diagnosis between this disease and other cardiomyopathies with hypertrophic phenotype (mainly HCM and cardiac amyloidosis) is crucial. The integration of CMR data with clinical examination, family history, ECG, and echocardiographic analysis guarantees the best accuracy in discriminating FD from other forms of LVH. However, the discussion of the multimodality approach to differential diagnosis [59, 60] is beyond the scope of this document.

Despite the wide range of hypertrophic phenotypes in FD cardiomyopathy, the concentric LVH is the most common pattern [21] as well as in cardiac amyloidosis, while in HCM hypertrophy is usually asymmetrical. Increased myocardial trabeculation is another feature of FD [22] but it has also been described in HCM. RV hypertrophy in FD correlates with LVH but, unlike cardiac amyloidosis, it does not significantly affect RV systolic function [56]. Combining the morphological pattern with tissue characterization increases the possibility of differentiating FD from others forms of LVH. As previously reported, low myocardial T1 values completely discriminate FD-related LVH from other cardiomyopathies such as HCM and cardiac amyloidosis [15]. Also, the coexistence of low T1 values with intramyocardial LGE in the basal infero-lateral wall should increase the suspicion of FD cardiomyopathy [34]. Indeed, HCM usually shows mid-wall LGE of anterior and posterior RV insertion points and hypertrophied segments while in amyloidosis LGE has a global subendocardial distribution (non-coronary pattern) or transmural [60].

The definite diagnosis of FD is based on the identification of a causative mutation in the *GLA* gene. Caution should be used in applying CMR in the study of *GLA* mutation with unknown significance, as no data are currently available regarding this application. Moreover, it is important to consider that CMR findings are a surrogate of histology and cannot replace the role of endomyocardial biopsy in specific settings.

## 7. Monitoring the Effect of Specific Therapies

By combining the accuracy in LV mass quantification and the multiparametric tissue characterization, CMR is the ideal tool for monitoring small changes over time in FD cardiac involvement. Previous CMR studies reported the reduction in LV mass and wall thickness in small populations after treatment with both the formulations of ERT (agalsidase α and β) [61, 62, 63, 64]. LGE has been recognized as a marker of advanced cardiac damage and a predictor of poor response to ERT. A recent study by Nordin *et al*. [65] is the only one to apply mapping techniques for the evaluation of the effect of ERT, stratifying patients according to the degree of cardiac involvement and pharmacological history. Twenty patients starting ERT were compared with 18 treatment-naïve patients with early disease, and 18 ERT patients with advanced disease. Over 1 year, the early disease treatment- naïve group showed increased maximum wall thickness and LV mass index and reduced native T1, reflecting the natural history of cardiac damage. In the advanced disease ERT group, an increase in T2 in LGE areas with worsening of global longitudinal strain was observed. Newly treated patients had a small reduction in maximum wall thickness and a reduction in T1 lowering, likely due to the effect of ERT in reducing myocardial glycosphingolipid storage.

## 8. Conclusions

In the past few years, CMR has been emerging as the ideal technique to study the complex pathophysiology of heart involvement in FD combining myocardial storage, hypertrophy, inflammation, and scarring. The application of CMR provided a powerful tool for the management of the most challenging clinical aspects, such as differential diagnosis from other forms of LVH, early detection of cardiac damage to promptly start specific treatment, and monitoring of the effects of therapies. Larger longitudinal studies are needed to define the prognostic values of CMR findings. The sensitivity and specificity of such parameters should be also defined by further histological studies.

## Abbreviations

CMR, Cardiac Magnetic Resonance; FD, Fabry Disease; LVH, Left Ventricular Hypertrophy; LV, Left Ventricle; ERT, Enzyme Replacement Therapy; LGE, Late Gadolinium Enhancement; ECV, ExtraCellular Volume fraction; HCM, Hypertrophic CardioMyopathy; LA, Left Atrium; RV, Right Ventricle; GLS, Global longitudinal strain; SAM, Systolic Anterior Motion; ECG, Electrocardiogram; PET, Positron Emission Tomography; FDG, Fluorodeoxyglucose.
